# Continuation of Mr. Littleton's Paper on the Eye and Vision

**Published:** 1816-10

**Authors:** 


THE LONDON
### Medical and Physical Journal.

4 OF VOL. XXXVI.] OCTOBER, 1816. [NO. 212.

"For many fortunate discoveries in medicine, and for the detection of nume-
"rous errors, the world is indebted to the rapid circulation of Monthly
"Journals; and there never existed any work to which the Faculty in
"EUROPE and AMERICA were under deeper obligations than to the
"Medical and Physical Journal of London, now forming a long, but an
"invaluable, series."—RUSH.

*For the London Medical and Physical Journal.*

### *Continuation of* Mr. LITTLETON's *Paper on the Eye and Vision.*

(See p. 89.)

### *Explanation of the Plates referred to in the former Paper.*

THE distance between the ends of the tangent lines meeting at *aaaa* mark the size of the pupil. That part of the iris included between the extremities of the lines which meet at *a*, and the extremities of the lines which meet at *b*, is the central annulus of the iris, which is distinguished from the peripheral annulus either by a distinct difference in colour, as in fig. 1, 2; or by an irregularly circular line, differing in colour from the annuli, as in fig. 3, 4. In some irides, I have also remarked that each annulus, and the line separating them, are all of a distinct colour; and, in some dark eyes, the iris seems nearly similar in colour throughout, yet, by a more attentive examination, the difference between the peripheral and central annulus can be perceived. The peripheral annulus is included between the extremities of the lines meeting at *b* and the extremities of the lines which meet at *c*; it is rather wider towards the external canthus than in other parts: this is plainly perceptible in many eyes.

### *On the Causes which enlarge the Size of the Pupil;* by Mr. LITTLETON.

HAVING mentioned, in my last paper, those causes which diminish the size of the pupil, I now proceed to the second part of the subject.

### *On the Causes which enlarge the Size of the Pupil.*

These causes I shall treat on in the following order:— 1. Sympathy. 2. Voluntary muscular exertion. 3. Convulsion. 4. Belladonna, &c. 5. Cerebral affections. 6. Palsy. 7. Scrofula. 8. Exhaustion. 9. Death.

1. On the mutual sympathy of the irides.—When we are looking at the irides in a moderate degree of light, if one eye be suddenly closed, the pupilar space of the other will be observed to immediately increase in size. This has very properly been referred to a sympathy with the closed eye, the pupil of which will undoubtedly be dilated.

This single fact strongly leads one to believe that the contraction of the pupil is not dependant on the degree of light falling on the retina. When we also conjoin the observation that in some cases of complete amaurosis, the pupils may be affected by light and sympathy, it may seem more confirmed, especially as it seems doubtful whether the experiments which have been thought to prove the contrary of this opinion can ever be satisfactory. The experiments to which I allude will be guessed at by the following question: How can the whole of the iris be exposed to light, and no light be allowed to fall on the retina?

From the existence of this sympathy, those who are affected with cataracts can see better with one eye shut than with both open. And, in ophthalmia of one eye, when the iris is affected, much pain may arise by leaving the sound eye exposed to light, as the sympathetic connection with the inflamed eye will prevent its iris from ever being at rest.

This sympathy may vary in degree in different individuals. By age, as the iris gradually becomes less irritable, the sympathy is less evident. By injuries or diseases it may also be altered, or even destroyed. Attentive observers, who have frequent opportunities of examining the state of the iris, in the various conditions of the body, may hereafter, perhaps, be able to detect diseases, even by attending to the state of this sympathy.

2. The pupil enlarges, directly as the degree of voluntary muscular exertion.—Any young person, or one whose iris has a tolerably extensive range of contraction, may observe this, in a moderate degree of light, in any common reflector. Having first attended to the state of the pupil, whilst his muscles are rather relaxed, he should then voluntarily exert, as much as possible, such muscles as his position will allow, at the same time observing the pupil, it will be seen to enlarge very evidently. A moderate degree of light only best allows of the perfect observance of this enlargement of the pupil, as more light might possibly be present than is sufficient to cause the extreme of diminution in the size of the pupil; and the light which may be superfluous to such an effect might counteract the disposition to enlargement caused by muscular exertion. On the other hand, if too little light be present,—for instance, such a small degree as allows of the extreme of enlargement of the pupil, then also the effect of muscular exertion might not be perceived.

Any

Any one acquainted with these facts might not, perhaps, find it difficult to deceive another who is ignorant of them, with respect to the voluntary command of the iris. Wherefore, I think it may be doubted whether parrots have a direct voluntary command of the contractions of their irides.

3. During the involuntary contraction of the muscles, as in convulsion, the enlargement of the pupil is much greater than during voluntary muscular exertion, being nearly equal, in some instances, to that caused by belladonna.—This may be remarked during the tonic or clonic action of muscles, when the system is vigorous; and also during the involuntary and weak muscular actions, attended with exhaustion, from whatever cause induced. I have constantly remarked this in every case of convulsion in which I have attended to it, viz. in convulsions of children where the causes may be various; in epilepsy, hysteria, cerebral injury; during the recovery from asphyxia, caused by carbonic acid gas; in hydrocephalus acutus, &c. I have also remarked that in rabbits under the influence of different vegetable poisons, whenever convulsion occurred, the pupils immediately enlarged. And rabbits, during the stupor caused by opium, in which state their pupils were diminished in size, being roused so as to cause some voluntary exertion of their muscles, their pupils would instantly increase in size.

There are, however, cases in which action of the voluntary muscles is present, whilst the pupil is small. I have remarked it in the restless somnolency which prevails in hydrocephalus acutus, where the eyes were open, and the child constantly sawing the air with one hand, the other upper extremity being paralysed. This state I consider as one of profound stupor, conflicting with some very great bodily uneasiness.

I have also remarked, that those who have been much subject to convulsion, as in epilepsy or hysteria, have the central annulus of the iris of a much larger size than I have seen it in other persons,—the frequent enlargements of the central annulus having, at last, caused a permanent encroachment on the peripheral ring. By the enlargement of the pupil, therefore, feigned convulsion may be detected; and, by attending to the state of the central annulus, we may even judge whether a person has, or has not, been much subject to convulsion. These states of the pupil, as connected with general muscular action, have, I believe, been heretofore unobserved.

4. The effects of belladonna on the pupil are well known. Soon after its application, the pupil begins to increase in size, and, after a few minutes, it is fully enlarged. The iris

 does does not recover its natural state for several days, or even a week. In the enlargement of the pupil from this cause, the central annulus is not to be perceived; the peripheral annulus is also very much diminished in width. In my observations on the effects of light on the iris, in my former paper, I mentioned that in small degrees of light, the pupils enlarged *proportionally* as the central annulus diminished. From observations since, however, I have found, by very suddenly varying the light to a great degree, that the peripheral annulus diminishes in width, at the same instant with the central ring: this is more especially detected in such young people as have the central annulus of a small size, compared with the peripheral portion of the iris. The difference with respect to contractility in these two parts of the iris is, however, evident. The central part appears to gradually diminish from its central edge, progressively, till it entirely disappears; whereas the peripheral part of the iris, by sudden exposure to much light, can be seen to decrease, by a contraction in its substance, its central edge remaining constant, although it may have approached to within half its usual distance from the corpus ciliare.

In the next place, I have to notice the effects of belladonna on vision, as ascertained by applying it to one eye only, by which means a comparison with the other eye is afforded. By viewing objects with the affected eye only, I found that its telescopic power still remained; and objects were seen equally distinct, but appeared much smaller in size, than when viewed with the unaffected eye. Objects viewed with both eyes at once were less distinct, and soon appeared confused, with a consequent feeling of fatigue in the eye-balls. By suddenly closing the affected eye, I observed that the iris of the other sympathized, and its pupil enlarged, as when both eyes were in their natural state. Also, before the belladonna has fully enlarged the pupil, it may be observed to increase in size, on shutting the lids of the unaffected eye. With the affected eye, letters could be read at the distance of 4 to 6 inches. These same letters were equally legible to the unaffected eye when only removed 2 inches to 2½ in., at which distance hazy lines only, instead of letters, could be discerned with the affected eye. To the affected eye, letters again ceased to be legible, when removed 16 to 20 in. whereas they could be read at the distance of 24 to 32 in. with the unaffected eye.

5. By cerebral affection, may be meant every case in which the brain is particularly deranged, whether from external violence or internal disease. As this, in many instances, is a general cause of other states of the body, which

I have

I have already treated on as causes affecting the size of the pupil, e. g. stupor, convulsion, &c.: it only remains, in this place, to mention any difference in the state of the pupil, arising from this cause, unconnected with the effects of stupor, convulsion, &c.

If, by any mechanical violence, or internal disease, those nerves which are distributed to the iris should have their functions interrupted, as by pressure, or when such nerves happen to be entirely divided, it is not to be always expected that the pupils would be small in stupor, or, perhaps, they might not enlarge in convulsion.

In phrenitis, I have not had opportunities of examining the irides. I am aware that the pupil is generally allowed to be diminished in size, but sufficient attention has not been paid to the many causes which influence it. I should, however, think it very likely, during the intolerance of light, that the irides might partake of the irritability of the retinæ, and that, therefore, light might more powerfully affect it; but, if there should be much consequent effusion within the cranium, or the strength should become much exhausted, it might give rise to an opposite state of the pupil.

During cerebral oppression, the diminished size of the pupil in sleep is less than in health, as in the following case:—A little boy, æt. four years, received a wound in the scalp, and had the right parietal bone fractured, by falling on a stone. A portion of the bone exfoliated, and the pulsation of the brain could afterwards be felt through the scalp. I am well informed that he had no symptoms of injury to the brain for four or five weeks, when he was attacked with convulsion on the left side of the body. Similar attacks recurred several times, at intervals of two to four weeks. After a few attacks, the left side of the body became paralysed; and then the right side had attacks of convulsion occasionally. Now and then, during the period of nine months following the injury, he has been fretful and peevish, with loss of appetite, flushed face, disturbed sleep, palpitations, frequency of micturition, &c. and, if neglected a few days, the bowels become obstinately costive, with much stupor. In this state of oppression, I thought the pupils were larger during sleep, which was rather profound, than when free from these attacks. The pulse, during sleep in these attacks, was irregular, or even intermittent. On waking, with increased quickness, it became regular. Calomel, in sufficient quantity to act on the bowels, soon restores him to his usual health and liveliness.

6. Palsy is another cause. But here we must not include those cases of palsy which arise from a mechanical cause, as these these more properly belong to the last division of the subject. Paralysis sometimes arises from causes which dissection can never detect—a gradual loss of power in the nerves themselves. This may be accompanied with loss of power in the retina also, or the retina may still retain some power, as in a case mentioned in my last paper. Although in amaurosis the pupils are most frequently dilated, the contrary state of the pupil sometimes exists in this disease, when no adhesion of the irides is at all probable: as no inflammation may have preceded, it may have arisen too suddenly to be accounted for as an effect of inflammation, or the diminution of the pupil may have come on gradually. Such diminished immoveable states of the pupil may be considered as arising from palsy of the radiated, or perhaps from tonic spasm of the circular fibres, with as much propriety as its enlarged immoveable state can be referred to palsy of the circular or tonic spasm of the radiated fibres of the iris.

7. A dilated pupil is commonly said to be present in scrofulous subjects.—As this is a disease of the fair, delicate, and weakly, who are proportionably irritable, it is not to be wondered at that their irides should partake of the general irritability of the system. One cause, however, why this dilatation may be observed is, that in such persons the black appearance of the pupil being contrasted with light-coloured irides, the size of the pupil more readily attracts our notice than in those who have their irides of a dark colour. In such persons as may be considered scrofulous, I have remarked thin light-coloured irides have either large or small central annuli. In some the central nearly resembles the peripheral annulus; in others it does not. I, therefore, doubt whether there may be any peculiar appearance plainly observable in the irides of scrofulous subjects, different from what may be observed in the irides of many others free from this disease.

8. In exhaustion, also, the body gradually loses its irritability, in consequence of which the range of contraction in the iris diminishes.—By the range of contraction I mean the difference between its extreme diminution and extreme enlargement. This difference, as the body becomes exhausted, gradually lessens. This gradual lessening of the range of contraction in the iris may be observed in such as die from protracted fevers, &c. in whom the iris, during sleep, is found more or less large, accordingly as is the degree of exhaustion. This same state I have also remarked in animals. Rabbits, soon after giving them some aqueous solution of opium, become listless, and remain in one position, with with their lids half closed, and the pupils diminished in size. In this state of stupor, by observing their pupils at intervals of one or two hours, I found they became gradually more enlarged as the vital powers became exhausted.

9. In death, I should expect to find the irides of a greater or less size, accordingly as the cause of death may have been sudden or gradual. If gradual exhaustion has preceded death, then an enlarged pupil would most probably be observed; but, if it should have been caused suddenly, as by mechanical injury of the brain, &c. I should not think it improbable to find the irides of a very diminished size. Perhaps the state of diminution or enlargement of the irides in death may sometimes depend on the state they are in immediately preceding death, as whether influenced by much light, sleep, stupor, convulsion, &c.*

I believe it is the generally-received opinion that in death the pupils are dilated. The term *dilated*, as generally used by practitioners, is undefined. It becomes a question, therefore, whether it means that the pupils are or are not so much enlarged as to obliterate the central annulus, and to what degree, beyond this annulus, the pupils are enlarged. In a woman, æt. 80, who died suddenly, as I conceive, from hæmorrhage near the heart, I observed the pupils did not occupy the whole of the central annulus. The state of the pupil, however, that I least expected to observe, after death, was, in some rabbits, six weeks old, in which the pupils were of very small, although not always of equal, size, even in the same rabbit. This diminished size of the pupil in rabbits occurred even when killed by belladonna, which dilates their pupils to an extreme degree, till the instant of death, when they immediately become decreased in area. The nature of the animal, as well as the causes of death, seems, therefore, to influence the state of the pupils.

I now, for the present, quit the subject, and hope I have not been so minute as to appear frivolous to all.

*Padstow; August*, 1816.

P.S.—If the illustrations are printed, the following observations may be subjoined to it:—The central and peripheral annuli cannot be distinctly perceived in children for many days after their birth. The iris, at first, appears hazy, and its colours blended together; but, as soon as the iris becomes clearer, and its striated appearance is more visible, then the exact line of distinction between the annuli can also be perceived. The first formation of the central annulus, as well as of the elliptical pupil, is most beautifully ob-

---

* We believe some of the French pathologists have observed the pupil contracted after death from hydrophobia.—Edit.

served in kittens: at first the irides are of a bluish colour, the pupil circular, and the central annulus scarcely perceivable; gradually the pupil becomes elliptical, the peripheral annulus almost entirely disappears, and a light-coloured central annulus, of an appearance resembling satin, occupies its place.—Having mentioned the ellipticity of the pupil in cats, I cannot forbear mentioning also the great utility of this shape of the pupil in these animals. As it is very necessary for them to see objects in small degrees of light, their pupils have the greatest possible range of contraction; and this is effected, not by an additional irritability of the iris, but by the direction of its fibres only, which fibres are thereby allowed to act more effectually in diminishing the aperture of the iris, inasmuch as the circumference of an oval bears a greater proportion to its area than the circumference of a circle does to the space it circumscribes.

---

*For the London Medical and Physical Journal.*

